# Analysis of healthcare needs differences and influencing factors among elderly population: Evidence from Yangtze River Delta region, China

**DOI:** 10.3389/fpubh.2022.949468

**Published:** 2022-09-26

**Authors:** Chen Li, Jiaji Wu, Yang Li, Yi Huang

**Affiliations:** ^1^School of Management, Shanghai University of Engineering Science, Shanghai, China; ^2^College of Humanities, Donghua University, Shanghai, China; ^3^School of Geographic Sciences, Nantong University, Nantong, China

**Keywords:** healthcare needs differences, comprehensive evaluation, influencing factors, Yangtze River Delta region, China

## Abstract

The quality of healthcare services is related to the quality of life of older people in their later years. A comprehensive evaluation of the healthcare needs of the elderly is the basic basis for providing targeted healthcare services for the elderly population. Taking the Yangtze River Delta region of China as an example, this article constructs an index system for evaluating the healthcare needs among the elderly, and explores the healthcare needs of the elderly and the influencing factors based on Dataset of the Fourth Sample Survey on the Living Conditions of China's Urban and Rural Older Persons. The study concludes that: Age 75 is the cut-off point for the healthcare needs of the elderly, with the growth of healthcare needs of the elderly aged 60–75 relatively flat and the growth of healthcare needs of the elderly aged 75 and above rising sharply. There is a wide variation in the Daily Activities Care Index, Incontinence Index and Aids Use Index scores and their indicators, as reflected in the differences in healthcare needs of older people in different age groups and in urban and rural areas. Healthcare needs of the elderly show a high positive correlation with the Daily Activities Care Index and Incontinence Index; healthcare needs of the elderly show a cubic function curve correlation with the assistive device use index. Community elderly healthcare services are conditions that influence the demand for elderly healthcare, but the low proportion of elderly people staying in elderly care institutions is due to a combination of low affordability, general quality of elderly care services and cultural factors. Educational attainment and marital status are micro-conditions that influence the demand for healthcare in old age. In terms of educational attainment, elderly people who have not attended school have an increasing need for healthcare as they get elderly. In terms of marriage, there is a strong negative correlation between the willingness to healthcare needs among elderly people with a spouse and a strong willingness to healthcare needs among elderly people who are widowed.

## Introduction

Aging is both a sign of rising life expectancy per capita and social progress, and a challenge for society. Life expectancy per capita in China in 2020 is 77.93 years. At the same time, the rapid increase in the elderly population poses a huge challenge to society. Data from the Seventh Population Census shows that in 2020, China will have 260.02 million elderly aged 60 years and above, accounting for 18.70% of the total population, and 190.64 million people aged 65 years and above, accounting for 13.50% of the total population, with the proportion of people aged 60 years and above rising by 5.44 percentage points compared to 2010 (1). According to forecasts, the size of China's elderly population will reach 412 million in 2035, and the number of senior citizens aged 80 and above will reach 61 million and 110 million in 2035 and 2050, respectively, with China maintaining the world's largest senior population for some time to come (2). Accompanying rapid aging is the issue of healthcare for the elderly, which has led to an increasing demand for health care among the elderly due to the decline in physiological functions and a significant increase in disease rates. With the combined impact of an aging population, advanced aging, empty nesting families and the weakening of traditional elderly care functions, the issue of health care services for a large number of empty nesters, the elderly who are left alone and disabled has become a focal point of concern for all sectors of society (3).

An overview of academic research on health care and its influencing factors in China's elderly population is as follows.

First, analysis of the current situation of healthcare services for the elderly. How to improve the detection rate of cognitive impairment among the elderly and ensure the continuity and effectiveness of long-term care for the elderly with cognitive impairment are the main risks and challenges facing care support for the elderly with cognitive impairment in China (4). The proportion of unmet care needs remained above 50% between 2005 and 2018; care resources tend to favor the elderly with moderate to severe disability, with the elderly with mild disability being the blind spot for care; although almost all elderly with moderate to severe disability have someone to care for them, they still need more care (5). In terms of development status, China's disabled and semi-disabled elderly population is growing rapidly and is unevenly distributed between urban and rural areas, with the probability of entering moderate and severe disability increasing with age and deteriorating initial health status in both urban and rural areas (6). The disability rate is higher than the incapacity rate in all age groups, but the incapacity rate increases more slowly then faster than the disability rate (7). The effect of time on the ability of older people to care for themselves has a random effect and is moderated by constant covariates over time (8). In terms of policy effects, although China has made great progress in the area of health care for the elderly, the policy on care for the disabled elderly faces many challenges, including imprecise policy positioning, lagging integrated policies, large differences in the level of care protection, inadequate coverage groups and responsible parties, and inadequate information on policy support recipients (9). The development of policies needs to be based on the analysis of the functional status of the elderly, the long-term care needs of the elderly and the future trends of the elderly based on the analysis of the elderly with disabilities and the elderly with dementia (10).

Second, analyses of the factors influencing healthcare for the elderly. Geriatric healthcare needs are diverse and multi-level, and they vary significantly among different age groups. The influence of healthcare for the elderly is influenced by a combination of many factors such as age, place of residence, education level and psychological status (11–13). Specifically, it is reflected in many aspects such as economic factors, policy factors, and social support. In terms of economic factors, the socio-economic status of Chinese elderly people is positively correlated with the receipt of appropriate medical care (14), and higher annual household income has a positive impact on the care of disabled elderly people in China (15). In terms of policy factors, public elderly care institutions are gradually developing from purely public welfare institutions to diversified investment and services (16), with problems such as low accessibility and underutilization of services, structural imbalance and system separation, insufficient supply capacity of private institutions, and multiple funding dilemmas (17). Due to the problems in the positioning of functions, operational mechanisms and resource allocation of public elderly institutions, the development of public elderly institutions is not dynamic enough (18, 19). Social support has been shown to be related to the health status of elderly people, with living with family members more likely to have higher levels of self-rated health (20). The impact of social support on the mental health of urban and rural elderly people is markedly different, with social support having a significant impact on the mental health of urban elderly people but not rural elderly people (21), and the most important factor limiting healthy aging in China's rural empty nesters is not physical health but social participation (22). In terms of health status, elderly people with chronic illnesses are at higher risk of mental disorders, particularly depression. Respondents who were female, on a low income, had a disability, lived in a rural area and were not working were more likely to have depressive symptoms. Conversely, those with increasing age, social insurance and good education were protective (23).

In summary, the above studies provide useful references for research on healthcare for the elderly, but there are also some areas that deserve further exploration. Most studies have examined the issue of healthcare for the elderly from the perspective of the supply side, and there is insufficient research on the evaluation of the demand side of the elderly, and there is a lack of indicator system construction and empirical analysis for the evaluation of the healthcare needs of the elderly population. Therefore, we take the Yangtze River Delta region of China as an example to evaluate the differences in healthcare needs of the elderly population and the influencing factors.

The above studies provide useful references for elderly health care research, but most studies examine the issue of elderly health care from a supply-side perspective, with little research on the differences in demand for elderly healthcare, and even less on the differences in demand for elderly health care between urban and rural areas, and a lack of analysis of the factors influencing the demand for elderly healthcare in urban and rural areas. Based on this, the study takes the Yangtze River Delta (YRD) region of China, a representative region in terms of aging, as an example, and analyses the difference in demand for elderly health care and its influencing factors between urban and rural areas in the YRD region. Healthcare for the elderly in this study refers to long-term care services due to physical incapacity, incontinence and physiological decline. The study will construct a comprehensive healthcare index to reveal the urban-rural differences in health care for the elderly in the Yangtze River Delta region in terms of three dimensions: daily activities, incontinence analysis and use of assistive devices, which will provide an important basis for accurately grasping the resource demand for care services for the elderly population and scientifically predicting and planning future elderly care institutions. The article is divided into six parts: Part 1 is an introduction, Part 2 is an overview of the study region and data sources, Part 3 is the research methodology and indicator construction, Part 4 is an evaluation of the healthcare needs of the elderly population, Part 5 is an analysis of the factors influencing the healthcare needs of the elderly, and Part 6 is discussions and conclusion.

## Study area and data sources

### Overview of the study area

**Figure 1:**
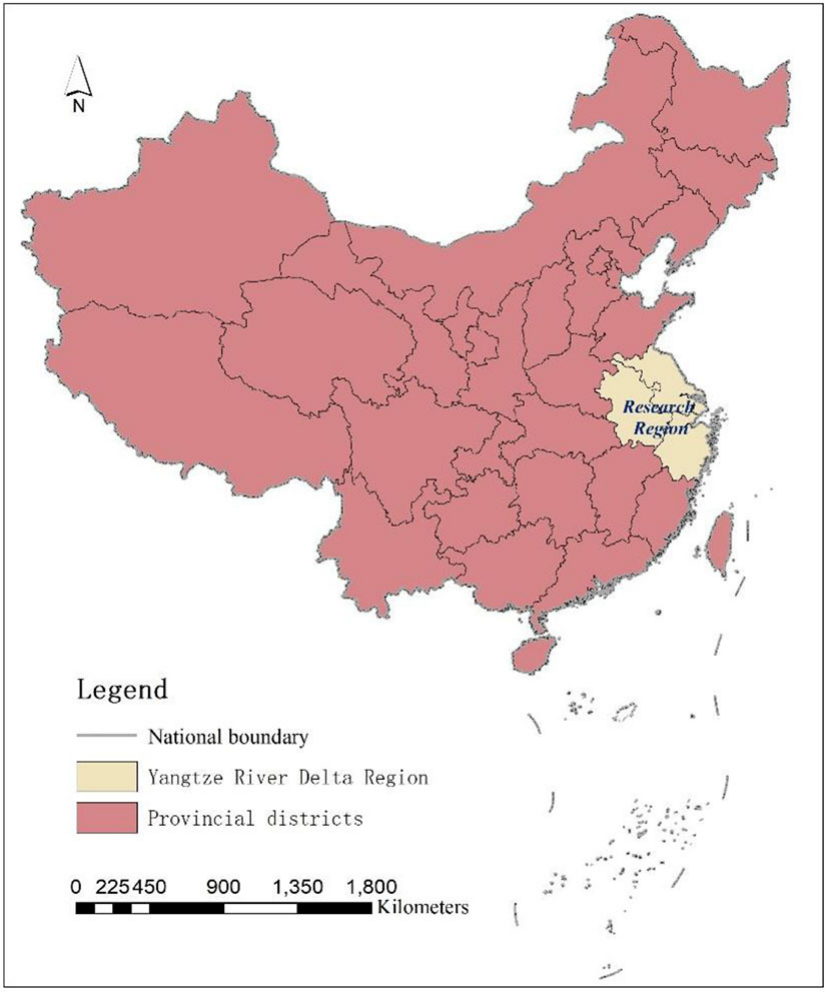
Research region.

The Yangtze River Delta region is located in the lower reaches of the Yangtze River basin, an alluvial plain formed before the Yangtze River enters the sea, and belongs to the subtropical humid monsoon climate zone. Administratively, the Yangtze River Delta region includes three provinces and one city, including Jiangsu Province, Zhejiang Province, Anhui Province and Mega-city Shanghai, with a land area of 358,000 km^2^ and is one of the most livable and densely populated regions in China (24–27), accounting for 16.75% of China's population size (Figure 1); GDP exceeds RMB 27.61 trillion, accounting for 24.14% of China's total economy; the urbanization rate of the resident population exceeds 60%, making it one of the six world-class urban agglomerations, the center of gravity region of China's industrial chain, and one of the most developed and active regions of China's economy (28).

As a result of improvements in people's living standards and healthcare in the Yangtze River Delta region, life expectancy per capita has continued to rise, while fertility rates continue to remain low, exacerbating the extent of population aging in the region. According to data from the 7th National Population Census (29–32), in 2020, the size of the elderly population aged 60 and above in the Yangtze River Delta region was ~47.87 million, accounting for 20.35% of the population. Among them, the size of the population aged 60 and above in Jiangsu Province is 185,053,345, accounting for 21.84%; the size of the population aged 60 and above in Shanghai is 58,195,580, accounting for 23.40%; the size of the population aged 60 and above in Zhejiang Province is 12,072,684, accounting for 18.70%; the size of the population aged 60 and above in Anhui Province is 11,469,236, accounting for 18.79%.

### Data sources

The data for this study were obtained from the Dataset of the Fourth Sample Survey on the Living Conditions of China's Urban and Rural Older Persons (Jiangsu Sub-volume, Shanghai Sub-volume, Zhejiang Sub-volume and Anhui Sub-volume), compiled by the China National Committee on Aging (33–36). The fourth sample survey on the living conditions of the elderly in urban and rural areas in China was conducted from 1 August to 31 August 2015. The data of the sample survey were collected through a network of civil affairs and aging agencies, such as “provinces and cities—districts (counties)—streets (towns)”, using a “stratified, multi-stage PPS, last-stage equal probability” approximate self-weighted sample sampling design. Taking into account the current dual economic structure between urban and rural areas in China, the fourth survey was designed in an urban-rural split, which provided a solid data base for our study of healthcare disparities between the urban and rural elderly populations.

There were 41,041 elderly respondents in the Yangtze River Delta region, with 38.58, 23.39, 10.48, and 27.55% of the sample from Jiangsu, Zhejiang, Shanghai, and Anhui, respectively. In terms of gender structure, 48.63% of the elderly respondents were male and 51.37% were female in the Yangtze River Delta region; in terms of education level, 39.78, 33.44, 16.23, 6.96, 2.23, and 1.36% had not attended school, primary school (including literacy classes), junior high school, high school/junior college/vocational high school, undergraduate level, and bachelor's degree and above, respectively. On marital status, 63.37, 32.46, 2.84, and 1.33% were spouse, widowed, divorced and never married, respectively; on health self-assessment, 7.07, 30.04, 40.75, 18.11, and 4.03% considered their health to be very good, relatively good, average, poor and very poor, respectively.

## Research methodology and indicator construction

### Methods

#### Healthcare composite index

The Healthcare Composite Index consists of Daily Activities Care Index (DCI), the Incontinence Index (ICI) and Aids Use Index (AUI), which reflect the level of healthcare needs of elderly people. Daily Activities Care Index (DCI), Incontinence Index (ICI) and Aids Use Index (AUI) reflect the level of healthcare needs of elderly people in a particular area. The higher the value of these three indices, the greater the level of healthcare needs of the elderly. The healthcare index was constructed by combining the characteristics of the sample data.

① Daily Activities Care Index, which is measured by the formula


(1) $$\begin{array}{l} DCI=\left(\sum \frac{u_{Dj}}{U_{Dj}}\times f_{i}\right)/n \end{array}$$

In the above equation, *u*_*Dj*_ is the sample size of an indicator of Daily Activities Care checked by elderly people in a certain age group; *U*_*Dj*_ is the total sample size of an indicator of daily activities care checked and unchecked by elderly people in a certain age group. *j* indicates the checkbox option, and there are three checkbox options in this study, namely “can do”, “some difficulty” and “can't do”. *f*_*i*_ assigns values to the ticked options, taking into account the characteristics of the sample data, *n* indicates the number of indicators in the Daily Activities Care Index (DCI), which in this study is 6. *DCI* is the Daily Activities Care Index and the higher the value, the greater the need for daily activities care.

② Construct Incontinence Index, which is measured by the formula


(2) $$\begin{array}{l} ICI=\frac{\sum 1/\left(u_{I}/U_{I}\right)}{n} \end{array}$$

In the above equation, *u*_*I*_ is the sample size of elderly people in an age group who ticked an option in the Incontinence Index; *U*_*I*_ is the total sample size of elderly people in an age group who ticked and unticked an option in the Incontinence Index. *n* indicates the number of indicators in the Incontinence Index, which in this study is 3. *ICI* is the Incontinence Index and the higher the value, the greater the degree of incontinence.

③ Construct Aids Use Index, which is measured by


(3) $$\begin{array}{l} AUI=\frac{\sum 1/\left(u_{A}/U_{A}\right)}{n} \end{array}$$

In the above equation, *u*_*A*_ is the number of elderly people in an age group who ticked an option in the Aids Use Index; *U*_*A*_ is the total number of elderly people in an age group who ticked and unticked an option in the Aids Use Index. *n* indicates the number of indicators in the Aids Use Index, 12 indicators in this study. *AUI* is the Aids Use Index, the higher the value, the greater the demand for aids. The higher the value, the greater the demand for the aid.

④ Construct Healthcare Composite Index, which is measured by


(4) $$\begin{array}{l} HCCI=DCI+ICI+AUI \end{array}$$

In the above equation, HCCI is the Healthcare Composite Index, *DCI, ICI* and *AUI* are the Daily Activities Care Index, Incontinence Index and Aids Use Index, respectively. The higher the value of *HCCI*, the higher the healthcare needs of the elderly.

#### Coefficient of variation

The coefficient of variation, also known as the standard deviation coefficient, is a measure of the degree of variation or dispersion of the sign values of the units of the total, and is meant to be the relative number of the standard deviation of the units of the total compared to their arithmetic mean. Where the standard deviation is the square root of the arithmetic mean of the sum of the squared deviations of the sign values of the units of the overall population from their arithmetic mean. The standard deviation is usually expressed as σ and the coefficient of variation as *CV*. The formula for measuring the standard deviation is (37).


(5) $$\begin{array}{l} \sigma =\sqrt{\frac{\sum {\left(x-\bar{x}\right)}^{2}}{n}} \end{array}$$

In the above equation, σ represents the standard deviation, *n* represents the number of samples, *x* represents the sign value of each unit and $\bar{x}$represents the mean of the sign values of each unit.

The formula for measuring the coefficient of variation is


(6) $$\begin{array}{l} CV=\frac{\sigma }{\mu }=\sqrt{\frac{\sum {\left(x-\bar{x}\right)}^{2}}{n}}/\mu \end{array}$$

In the above equation, *CV* represents the coefficient of variation, σ represents the standard deviation and μ represents the arithmetic mean.

We will use the coefficient of variation to measure differences in the healthcare needs of the elderly population between different age groups and urban and rural areas to reveal the characteristics of the differences in their healthcare needs by age group and urban and rural areas.

#### Correlation analysis

The scatter plot is the most visual method used to express correlation analysis. The correlation coefficient is a collective term for a class of indicators that measure the correlation between variables (38, 39).

The common correlations are linear correlation, curvilinear correlation, positive correlation, and negative correlation. The Person correlation coefficient, also known as the product-difference correlation coefficient, is a common metric for quantitatively describing the degree of linear correlation. The formula for measuring the Person correlation coefficient is


(7) $$\begin{array}{l} r=\frac{\sum \left(x_{i}-\overset{-}{x}\right)\left(y_{i}-\overset{-}{y}\right)}{\sqrt{{\left(x_{i}-\overset{-}{x}\right)}^{2}}\sqrt{{\left(y_{i}-\overset{-}{y}\right)}^{2}}} \end{array}$$

In Equation 7, *x*_*i*_ and *y*_*i*_ are the variables, $\bar{\text{x}}$ and $\bar{\text{y}}$ are the means of variables *x*_*i*_ and *y*_*i*_, and *r* is the correlation coefficient. The maximum correlation coefficient is 1. The closer the absolute value of the correlation coefficient is to 1, the stronger the correlation between the variables. Our study will measure the correlation coefficient between Geriatric healthcare needs and its influencing variables using correlation coefficients, and visually express the relationship between them by means of scatter plots.

### Indicator system construction

Activity of Daily Life (ADL) is a commonly used international measurement tool for assessing basic independent living and activity in older people and is the most important indicator of the health care needs of older people, with six assessment items including bathing, dressing, toileting, mobility, continence control and eating (40, 41). Although the ADL index is widely accepted and commonly used globally, its assessment reliability has been criticized due to the high subjectivity of respondents. Therefore, this study adds the evaluation of objective indicators, such as the use of aids, in addition to the design of common ADL indicators, to make its evaluation more completed. The core indicators of the comprehensive healthcare index consist of three aspects: Daily Activities Care Index, Incontinence Index, and Aids Use Index, with a total of 21 three-level indicators; the reference indicators consist of education level, marital status, and willingness to pay for moving into a nursing home, with a total of 16 options for these three indicators (Table 1). We will use the 21 three-level indicators to measure the Daily Activities Care Index, Incontinence Index and Aids Use Index respectively to reflect the healthcare needs of the elderly population in the Yangtze River Delta region and reveal the characteristics of their differences.

**Table 1:** Healthcare indicator system.

**Secondary indicators**	**Tertiary indicators**	**Options**
Daily activities care index	Meal situation	Can do/Some difficulties/Can't do
	Clothing situation	Can do/Some difficulties/Can't do
	Go to the toilet	Can do/Some difficulties/Can't do
	Get in and out of bed	Can do/Some difficulties/Can't do
	Walking around indoors	Can do/Some difficulties/Can't do
	Bathing situation	Can do/Some difficulties/Can't do
Incontinence index	Fecal incontinence	Yes/No
	Urinary incontinence	Yes/No
	Have incontinence	Yes/No
Aids use index	Use of presbyopia glasses	Yes/No
	Use of hearing aids	Yes/No
	Use of dentures	Yes/No
	Use of crutches	Yes/No
	Use of wheelchairs	Yes/No
	Use of blood pressure monitors	Yes/No
	Use of blood glucose meters	Yes/No
	Use of care pads	Yes/No
	Use of massage instruments	Yes/No
	Use of smart wear	Yes/No
	Use of care beds	Yes/No
	Use of other aids	Yes/No
Level of education	No schooling	Yes/No
	Primary schools (including literacy classes)	Yes/No
	Junior High School	Yes/No
	High School	Yes/No
	Undergraduate level	Yes/No
	Bachelor and above	Yes/No
Marital status	With a spouse	Yes/No
	Bereaved spouse	Yes/No
	Divorced	Yes/No
	Never married	Yes/No
Willingness to move into nursing home expenses	Less than $1000	Yes/No
	$1000–$1999	Yes/No
	$2000–$2999	Yes/No
	$3000–$3999	Yes/No
	$4000–$4999	Yes/No
	More than $5000	Yes/No

Daily Activities Care. This type of indicator reflects the basic activities of daily living of elderly people and provides a comprehensive picture of the level of need for daily activities care in terms of basic eating, dressing, toileting, getting in and out of bed, bathing and walking around indoors.

Incontinence. As we age, the sphincter muscle becomes relaxed and damaged due to aging, resulting in loss of control over bowel movements and incontinence. Incontinence has a serious impact on the quality of life of elderly people in their later years and is an important aspect of healthcare.

Aids Use. As they age, some elderly people are relying on aids such as presbyopia glasses, hearing aids, crutches, wheelchairs, care beds and nursing mats. On the one hand, the use of assistive devices indicates an increase in the level of healthcare for the elderly population; on the other hand, it is the use of assistive devices that has contributed to some of the elderly population becoming somewhat less dependent on others for care and partly self-managing their health.

Level of Education. Educational attainment has a significant impact on healthcare for elderly people, with the more educated elderly group being slightly better at smart dressing, healthy eating and scientific knowledge for their own healthcare.

Marital Status. Marital status plays an important role in the healthcare of elderly people in later life, as reflected in the mutual care of their partners.

The cost expenditure range for willingness to move into a nursing home. This indicator is mainly used for the analysis of the economic influences on healthcare in different elderly groups, which we will explain further in the text.

## Comprehensive assessment of healthcare needs among elderly population

### General analysis of healthcare needs among elderly population

This section provides a comprehensive evaluation of the healthcare needs of elderly people in the Yangtze River Delta region and the healthcare needs of elderly people in Jiangsu, Shanghai, Zhejiang and Anhui in the Yangtze River Delta region, respectively.

Firstly, a comprehensive evaluation of the healthcare needs of elderly people in the Yangtze River Delta region revealed three characteristics in terms of their healthcare needs (Table 2).

**Table 2:** Elderly healthcare index measurements in the Yangtze River Delta region.

**Indicators**	**Age group**	**Daily activities**	**Incontinence**	**Aids use**	**Healthcare**
		**care index**	**index**	**index**	**composite index**
**Region**					
Yangtze River Delta region	60–64	1.031	1.016	1.116	3.162
(urban and rural regions of	65–69	1.049	1.029	1.131	3.209
Jiangsu, Shanghai, Zhejiang, Anhui)	70–74	1.088	1.041	1.135	3.264
	75–79	1.162	1.066	1.142	3.370
	80–84	1.245	1.083	1.165	3.493
	85+	1.473	1.157	1.167	3.797
Yangtze River Delta region	60–64	1.027	1.012	1.137	3.175
(urban regions of Jiangsu,	65–69	1.038	1.019	1.163	3.219
Shanghai, Zhejiang and Anhui)	70–74	1.081	1.028	1.170	3.279
	75–79	1.146	1.055	1.180	3.381
	80–84	1.266	1.091	1.207	3.564
	85+	1.478	1.152	1.206	3.836
Yangtze River Delta region	60–64	1.036	1.022	1.090	3.148
(rural regions of Jiangsu,	65–69	1.065	1.042	1.097	3.204
Shanghai, Zhejiang and Anhui)	70–74	1.098	1.059	1.097	3.254
	75–79	1.184	1.081	1.100	3.365
	80–84	1.212	1.070	1.110	3.391
	85+	1.470	1.165	1.122	3.756

Characteristic one: the healthcare composite index of the elderly population in the Yangtze River Delta region gradually increases with age. The healthcare needs of the elderly aged 60–64 are relatively the smallest, and those aged 85 and above are relatively the largest. In terms of age structure, the healthcare needs of the three age groups of 60–64, 65–69, and 70–74 years old grew relatively slowly, with the Healthcare Composite Index increasing from 3.162 to 3.264, a growth rate of 3.25%, while the healthcare needs of the three age groups of 75–79, 80–84, and 85 years old and above grew relatively quickly, with the Healthcare The overall index increased from 3.370 to 3.797, with a growth rate of 12.67%.

Characteristic two: elderly people have a higher level of need for daily activities care than for incontinence care, and a higher level of need for incontinence care than for the use of aids. Compared to the 60–64 age group, the index of daily activities care expanded from 1.031 to 1.473, the index of incontinence from 1.016 to 1.157, and the index of aids use from 1.116 to 1.167 for the 85+ age group, with growth rates of 42.87%, 13.88%, and 4.57% for the three, respectively.

Characteristic three: there is little difference between the urban and rural elderly populations in the Yangtze River Delta in terms of incontinence care, but there are large differences in the need for daily activities care and the use of assistive devices. In terms of daily activities care needs, the growth rate of the DCI index is 43.91% in urban areas of the Yangtze River Delta and 41.89% in rural areas, with a difference of 2.02 percentage points between urban and rural growth rates. The growth rate of the ICI index is 13.83% in urban areas of the Yangtze River Delta and 13.99% in rural areas, with a difference of 0.16 percentage points between urban and rural areas; the growth rate of the AUI index is 6.07% in urban areas of the Yangtze River Delta and 2.94% in rural areas, with a difference of 3.13 percentage points between urban and rural areas.

Secondly, the healthcare needs of elderly people in the Yangtze River Delta region were evaluated in Jiangsu, Shanghai, Zhejiang and Anhui (Table 3).

**Table 3:** Aged healthcare composite index for Jiangsu, Shanghai, Zhejiang and Anhui.

**Region**	**Type**	**60–64**	**65–69**	**70–74**	**75–79**	**80–84**	**85+**
Jiangsu	U & R	3.130	3.171	3.236	3.341	3.459	3.725
Jiangsu	Urban	3.139	3.164	3.243	3.342	3.535	3.747
Jiangsu	Rural	3.115	3.186	3.226	3.346	3.330	3.715
Shanghai	U & R	3.191	3.241	3.320	3.461	3.596	3.862
Shanghai	Urban	3.211	3.266	3.321	3.472	3.633	3.902
Shanghai	Rural	3.079	3.142	3.338	3.457	3.440	3.672
Zhejiang	U & R	3.236	3.290	3.313	3.332	3.435	3.685
Zhejiang	Urban	3.254	3.312	3.347	3.354	3.513	3.854
Zhejiang	Rural	3.218	3.270	3.284	3.312	3.346	3.456
Anhui	U & R	3.157	3.212	3.280	3.443	3.565	4.011
Anhui	Urban	3.171	3.241	3.310	3.478	3.656	3.992
Anhui	Rural	3.151	3.200	3.267	3.425	3.488	4.044

Overall, the Healthcare Composite Index (HCCI) in Jiangsu, Shanghai, Zhejiang and Anhui all show a gradual increase with age, with the higher the age of the elderly population, the greater the level of healthcare needs. The coefficient of variation for 60–64, 65–69, and 70–74 years is below 0.015, but increases to 0.020 for 75–79 years and rapidly to 0.039 for 85 years and over.

In terms of urban areas, the demand for elderly healthcare in Jiangsu, Shanghai, Zhejiang and Anhui is gradually increasing, with the average HCCI index for urban areas in Jiangsu, Shanghai, Zhejiang and Anhui increasing from 3.194 in the 60–64 age group to 3.874 in the 85+ age group, an increase of 21.29 percentage points. The overall variation in the HCCI index for urban areas shows a fluctuating pattern of “up–down–up–down–up”, with a coefficient of variation of 0.016 for the 60–64 age group and 0.026 for the 85+ age group, representing a growth rate of 68.90%.

In terms of rural areas, the demand for healthcare in rural areas in Jiangsu, Shanghai, Zhejiang and Anhui is gradually increasing, with the average HCCI index in rural areas increasing from 3.141 in the 60–64 age group to 3.722 in the 85+ age group, an increase of 18.50 percentage points, slightly lower than in urban areas. The overall variation in the HCCI index in rural areas showed a “downward–upward” trend, with the coefficient of variation decreasing from 0.019 for the 60–64 age group to 0.014 for the 70–74 age group and then increasing to 0.065 for the 85+ age group, an increase of 3.46 times.

### Evaluation of secondary indicators of healthcare needs among elderly population

#### Analysis of daily activities among elderly population

This section will analyze the overall situation and urban-rural differences in the daily activities care of elderly people in Jiangsu, Shanghai, Zhejiang and Anhui, respectively.

**Figure 2:**
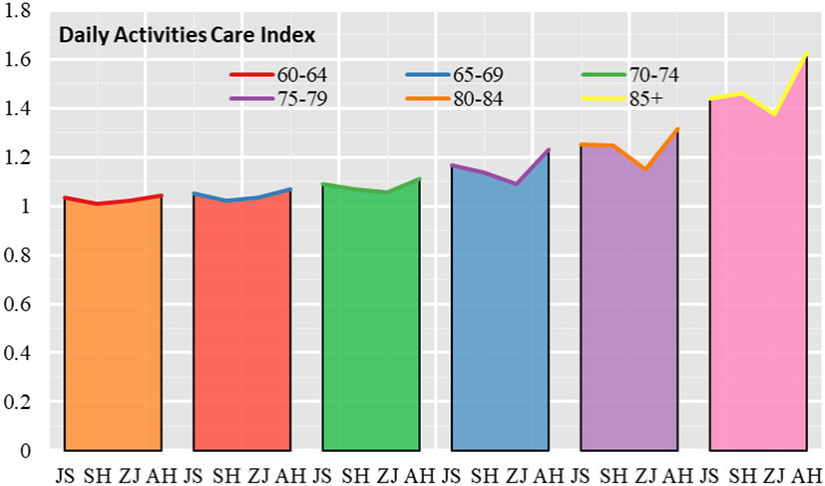
Daily activities care index for elderly people in Jiangsu, Shanghai, Zhejiang and Anhui.

In terms of the overall demand for daily activities care in Jiangsu, Shanghai, Zhejiang and Anhui, the demand for daily activities care for elderly people in Anhui is higher than that in Jiangsu, the demand for daily activities care for elderly people in Jiangsu is higher than that in Shanghai, and the demand for daily activities care for elderly people in Shanghai is higher than that in Zhejiang. The daily activities care indexes for elderly people in Jiangsu, Shanghai, Zhejiang and Anhui were 1.172, 1.158, 1.121, and 1.233, respectively. In terms of age groups, the need for daily activities care is greatest in the 85+ age group and least in the 60–64 age group (Figure 2). Looking at the difference in demand in Jiangsu, Shanghai, Zhejiang and Anhui, the difference in demand for daily activities care gradually widens from the 60–64 age group to the 85+ age group, with the coefficient of variation expanding from 0.014 to 0.073, a 5.28-fold increase in the coefficient of variation.

**Figure 3:**
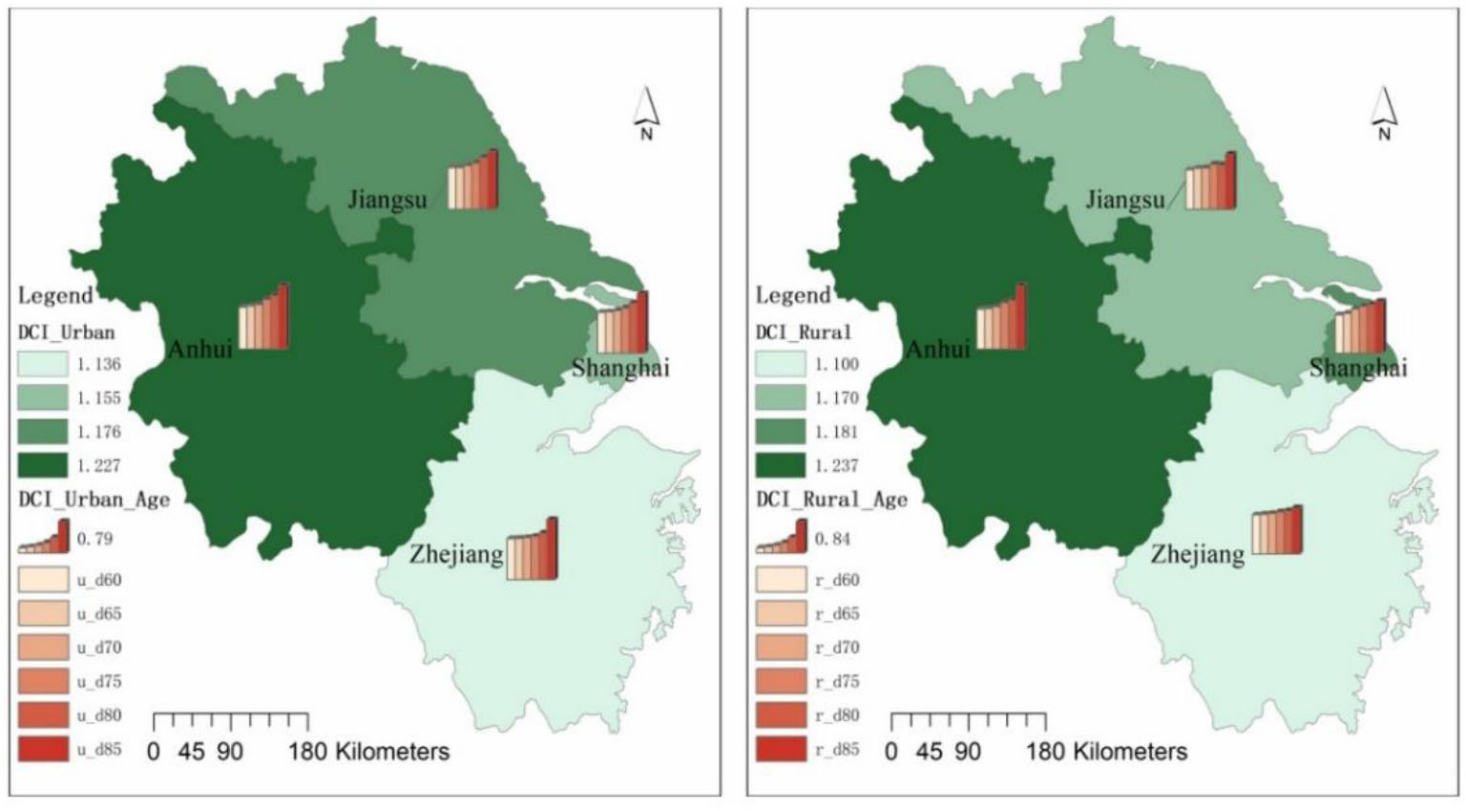
Daily activities care index for elderly people in urban and rural areas in Jiangsu, Shanghai, Zhejiang and Anhui.

Looking at the urban-rural differences in daily activities care in Jiangsu, Shanghai, Zhejiang and Anhui, the urban-rural differences in the daily activities care index began to increase significantly with the age of the elderly. In the 60–64 age group, 65–69 age group and 70–74 age group, the urban-rural differences in the daily activities care index were not significant, but in the 75–79 age group, 80–84 age group and 85+ age group the urban-rural differences increased significantly (Figure 3).

In terms of the coefficient of variation for each age group, the coefficient of variation for the Urban Daily Activities Care Index is 0.011 and the Rural Daily Activities Care Index is 0.020 in the 60–64 age group; the coefficient of variation for the Urban Daily Activities Care Index is 0.024 and the Rural Daily Activities Care Index is 0.014 in the 65–69 age group; the coefficient of variation for the Urban Daily Activities In the 70–74 age group, the coefficient of variation of the daily activities care index is 0.028 in urban areas and 0.036 in rural areas; in the 75–79 age group, the coefficient of variation of the daily activities care index is 0.057 in urban areas and 0.050 in rural areas; in the 80–84 age group, the coefficient of variation for the Urban Daily Activities Care Index is 0.060 and the Rural Daily Activities Care Index is 0.065; in the 85+ age group, the coefficient of variation for the Urban Daily Activities Care Index is 0.039 and the Rural Daily Activities Care Index is 0.135.

In terms of the scores of each provincial administrative unit, relatively speaking, Anhui has the largest urban-rural daily activities care index, which is much higher than the average of the Yangtze River Delta region; Zhejiang has the relatively smallest urban-rural daily activities care index, which is much lower than the average of the Yangtze River Delta region; Shanghai and Jiangsu have an urban-rural daily activities care index close to the average of the Yangtze River Delta region.

Comparison of the coefficients of variation of the tertiary indicators for daily activities care in Jiangsu, Shanghai, Zhejiang, and Anhui (Table 4).

**Table 4:** Coefficients of variation for indicators of daily activities care.

**Region**	**Type**	**Meal**	**Clothing**	**Go to the toilet**	**Get in and out of bed**	**Walking around**	**Bathing**
Jiangsu	U & R	0.103	0.116	0.147	0.126	0.092	0.225
Shanghai	U & R	0.086	0.128	0.136	0.135	0.145	0.255
Zhejiang	U & R	0.060	0.099	0.107	0.111	0.137	0.190
Anhui	U & R	0.119	0.140	0.185	0.167	0.185	0.258
YRD	U & R	0.094	0.120	0.146	0.134	0.116	0.230
Jiangsu	Urban	0.111	0.124	0.159	0.135	0.125	0.239
Shanghai	Urban	0.091	0.134	0.141	0.141	0.146	0.273
Zhejiang	Urban	0.084	0.141	0.144	0.155	0.180	0.244
Anhui	Urban	0.106	0.139	0.173	0.150	0.164	0.255
YRD	Urban	0.098	0.132	0.154	0.142	0.111	0.249
Jiangsu	Rural	0.093	0.106	0.132	0.117	0.127	0.203
Shanghai	Rural	0.064	0.099	0.119	0.119	0.156	0.166
Zhejiang	Rural	0.029	0.043	0.060	0.051	0.080	0.114
Anhui	Rural	0.133	0.142	0.196	0.184	0.207	0.263
YRD	Rural	0.089	0.103	0.136	0.124	0.144	0.202

At an overall level, Anhui has the largest variation in the indicators of daily activities for all age groups, as reflected in the coefficients of variation for the indicators of being able to eat, dress, go to the toilet, get in and out of bed, walk around indoors and bathe. In Zhejiang, the differences in the indicators of daily activities were relatively smallest, as reflected by the coefficients of variation in the three levels of indicators of daily activities. The coefficients of variation for Jiangsu and Shanghai are in the second and third positions.

At the city level, the city-level coefficients of variation for the ability to eat, dress, go to the toilet and get into and out of bed were higher than the overall coefficients of variation, while the city-level coefficients of variation for the ability to walk indoors and take a bath were lower than the overall coefficients of variation. At the city level, the coefficients of variation were highest for the ability to eat for each age group in Jiangsu, while the coefficients of variation were highest for the ability to dress, go to the toilet, get in and out of bed, move around indoors and take a shower for each age group in Anhui.

At the rural level, the coefficients of variation for the ability to eat, dress, go to the toilet, get in and out of bed, and bathe were lower at the rural level than at the urban level, and lower than at the overall level. Rural elderly people in the Yangtze River Delta have higher variation in the ability to walk around indoors than those in urban areas. As with both the overall and urban levels, the coefficient of variation is highest for the ability to bathe and lowest for the ability to eat.

### Analysis of incontinence among elderly population

This section will analyze the overall situation and urban-rural differences in the incontinence index for elderly people in Jiangsu, Shanghai, Zhejiang and Anhui, respectively.

**Figure 4:**
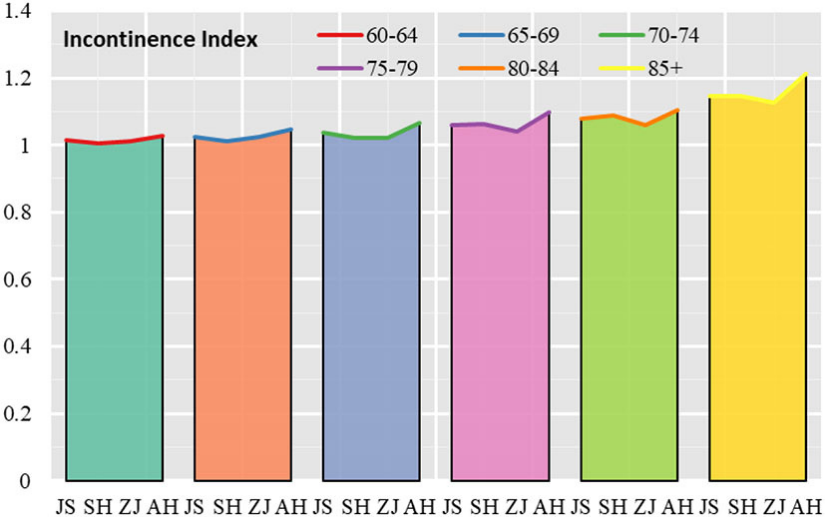
Incontinence index for elderly people in Jiangsu, Shanghai, Zhejiang and Anhui.

In terms of the overall situation, the incontinence index for elderly people in Anhui was higher than in Jiangsu, the incontinence index for elderly people in Jiangsu was higher than in Shanghai, and the incontinence index for elderly people in Shanghai was higher than in Zhejiang, with incontinence indices of 1.093, 1.060, 1.056, and 1.047 for elderly people in Anhui, Jiangsu, Shanghai and Zhejiang, respectively. The age group had the lowest probability of incontinence (Figure 4). In terms of variation in incontinence by provincial and municipal elderly age groups, elderly people in Anhui had the highest coefficient of variation in the three indicators of fecal incontinence, urinary incontinence and presence of incontinence by age group. The coefficients of variation for incontinence indicators by age group were 0.056, 0.066, 0.047, and 0.073 in Jiangsu, Shanghai, Zhejiang and Anhui, respectively, with the largest coefficient of variation in Anhui and the smallest in Zhejiang.

**Figure 5:**
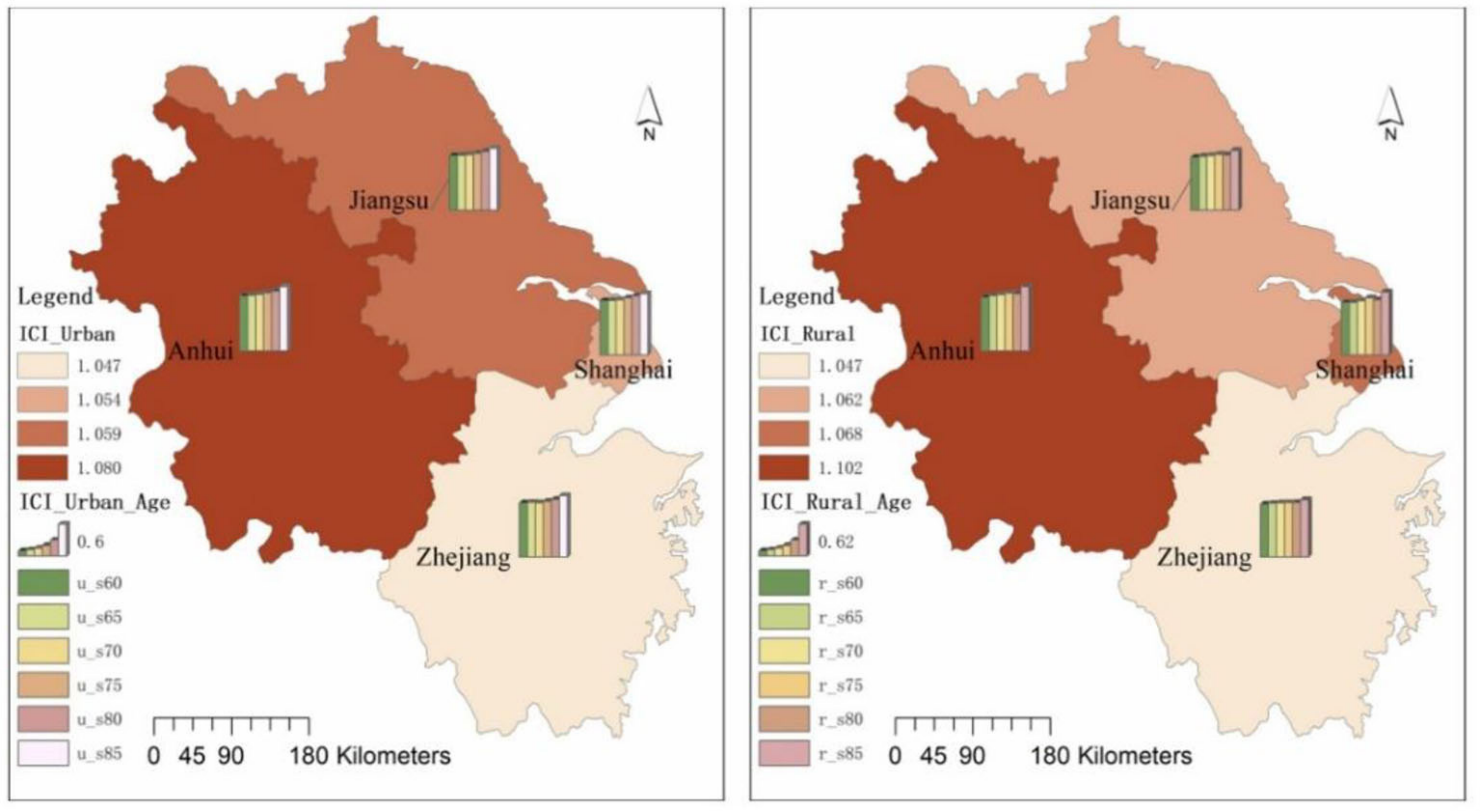
Urban and rural incontinence indices for elderly people in Jiangsu, Shanghai, Zhejiang and Anhui.

Looking at the situation in urban and rural areas, the incontinence index of the elderly in urban areas began to increase significantly with the increase in the age of the elderly, with the urban incontinence index of the elderly in the 60–64 age group, 65–69 age group, 70–74 age group, 75–79 age group, 80–84 age group, and 85+ age group being 1.012, 1.019, 1.028, 1.055,1.091, and 1.152. In rural areas, the incontinence indices for the 60–64, 65–69, 70–74, 75–79, 80–84, and 85+ age groups were 1.022, 1.042, 1.059, 1.081, 1.070, and 1.165, respectively. The above data reflect that the level of incontinence is higher in rural areas than in urban areas in the 60–79 age group. In addition to the 80–84 age group where urban elderly people are more incontinent than rural areas, rural elderly people in the 85+ age group are more incontinent than urban areas (Figure 5).

The incontinence indices for the urban and rural age groups were 1.015, 1.005, 1.007, and 1.018 for the urban elderly in Jiangsu, Shanghai, Zhejiang and Anhui in the 60–64 age group, and 1.016, 1.005, 1.017, and 1.032 for the rural elderly in Jiangsu, Shanghai, Zhejiang and Anhui. In 85+ age group, the incontinence index was 1.147, 1.136, 1.141, and 1.191 for the urban elderly in Jiangsu, Shanghai, Zhejiang and Anhui, respectively, and 1.144, 1.203, 1.104, and 1.232 for the rural elderly in Jiangsu, Shanghai, Zhejiang and Anhui, respectively.

In terms of the scores of each provincial administrative unit, relatively speaking, Anhui has the largest urban and rural elderly incontinence index, which is higher than the average of the Yangtze River Delta region; Zhejiang has the smallest urban and rural elderly incontinence index, which is lower than the average of the Yangtze River Delta region; Shanghai and Jiangsu have an urban and rural elderly incontinence index close to the average of the Yangtze River Delta region.

The coefficients of variation for the sub-indicators of incontinence care for elderly people in Jiangsu, Shanghai, Zhejiang and Anhui will be compared below (Table 5).

**Table 5:** Coefficient of variation of incontinence index indicators.

**Region**	**Type**	**Fecal incontinence**	**Urinary incontinence**	**Have incontinence**
Jiangsu	U & R	0.030	0.049	0.056
Shanghai	U & R	0.030	0.056	0.066
Zhejiang	U & R	0.035	0.039	0.047
Anhui	U & R	0.046	0.061	0.073
Yangtze River Delta	U & R	0.034	0.050	0.059
Jiangsu	Urban	0.033	0.052	0.060
Shanghai	Urban	0.029	0.055	0.066
Zhejiang	Urban	0.043	0.051	0.056
Anhui	Urban	0.042	0.062	0.073
Yangtze River Delta	Urban	0.035	0.054	0.063
Jiangsu	Rural	0.027	0.047	0.052
Shanghai	Rural	0.044	0.082	0.081
Zhejiang	Rural	0.025	0.024	0.037
Anhui	Rural	0.051	0.063	0.075
Yangtze River Delta	Rural	0.034	0.047	0.056

At an overall level, the greatest variation in the indicators of incontinence among elderly people of all age groups in Anhui is reflected in the coefficients of variation for the indicators of “fecal incontinence”, “urinary incontinence” and “incontinence”, which are 0.046, 0.061, and 0.073, respectively. The coefficients of variation for the indicators of “fecal incontinence” were 0.046, 0.061, and 0.073 for Shanghai and Jiangsu, respectively, while the coefficients of variation for the indicators of “urinary incontinence” and “incontinence” were 0.030 for Zhejiang. The coefficients of variation for the indicators of “urinary incontinence” and “incontinence” were relatively small in Zhejiang, at 0.039 and 0.047, respectively.

At the city level, the coefficient of variation for the indicator “incontinence” was the largest, followed by the coefficient of variation for “urinary incontinence” and the smallest coefficient of variation for “fecal incontinence”. The coefficients of variation for the indicator of “incontinence” were 0.060, 0.066, 0.056, and 0.073 for Jiangsu, Shanghai, Zhejiang and Anhui, respectively. The coefficients of variation for the indicator of “fecal incontinence” were 0.033, 0.029, 0.043, and 0.042 for the elderly in Jiangsu, Shanghai, Zhejiang and Anhui, respectively.

At the rural level, the coefficient of variation for the indicator of “fecal incontinence” was the largest in Anhui, at 0.051, while the coefficients of variation for the indicators of “urinary incontinence” and “incontinence” were the largest in Shanghai, at 0.082 and 0.081, respectively. The coefficients of variation for the indicators of “fecal incontinence”, “urinary incontinence” and “incontinence” were 0.082 and 0.081, respectively for the elderly in rural Zhejiang. The coefficients of variation for the three indicators of “fecal incontinence”, “urinary incontinence” and “incontinent” were the smallest, at 0.025, 0.024, and 0.037, respectively, which were much lower than the average level of variation in incontinence indices among the rural elderly in the Yangtze River Delta region.

### Analysis of aids use among elderly population

This section will analyze the use of Aids Use (or Assistive device use) for the elderly in Jiangsu, Shanghai, Zhejiang and Anhui, and the differences between urban and rural areas, respectively.

**Figure 6:**
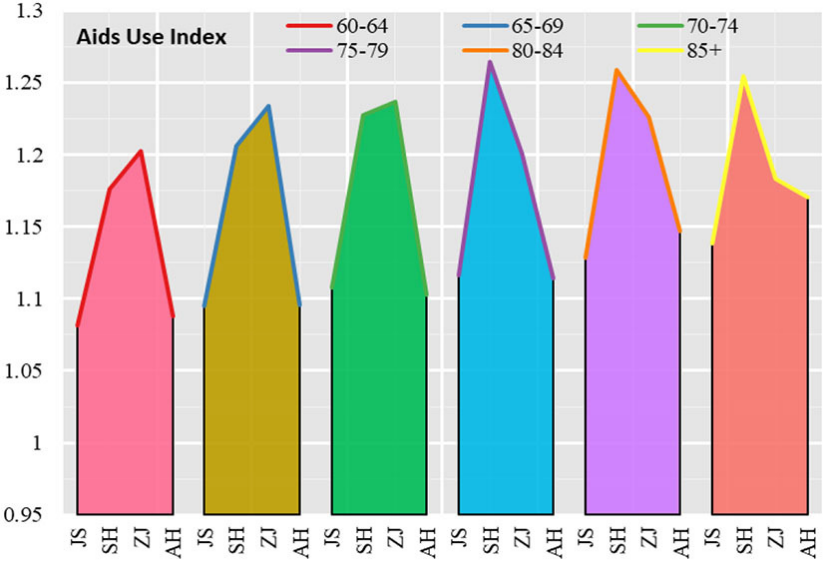
Aids Use index for elderly people in Jiangsu, Shanghai, Zhejiang and Anhui.

Overall, the use of assistive devices among elderly people in Shanghai was higher than that in Zhejiang, while the use of assistive devices among elderly people in Zhejiang was higher than that in Anhui and slightly higher than that in Jiangsu. In terms of age groups, the gradient in the use of assistive devices for elderly people in Shanghai and Zhejiang is not obvious as the age increases. Shanghai had the highest indicator of assistive device use in the 75–79 age group, while Zhejiang had the highest indicator in the 80–84 age group. In Jiangsu and Anhui, there is a clear gradient in the use of assistive devices with increasing age, that's, the probability of assistive device use gradually increases for the 60–64, 65–69, 70–74, 75–79, 80–84, and 85+ age groups. In terms of differences in the use of assistive devices by age group, the coefficient of variation of the index of assistive device uses among elderly people in the 60–64, 65–69, 70–74, 75–79, 80–84, and 85+ age groups show an “inverted U” shape, that's, the coefficient of variation of the index of elderly people's use of assistive devices is in a “rising–falling” process (Figure 6).

**Figure 7:**
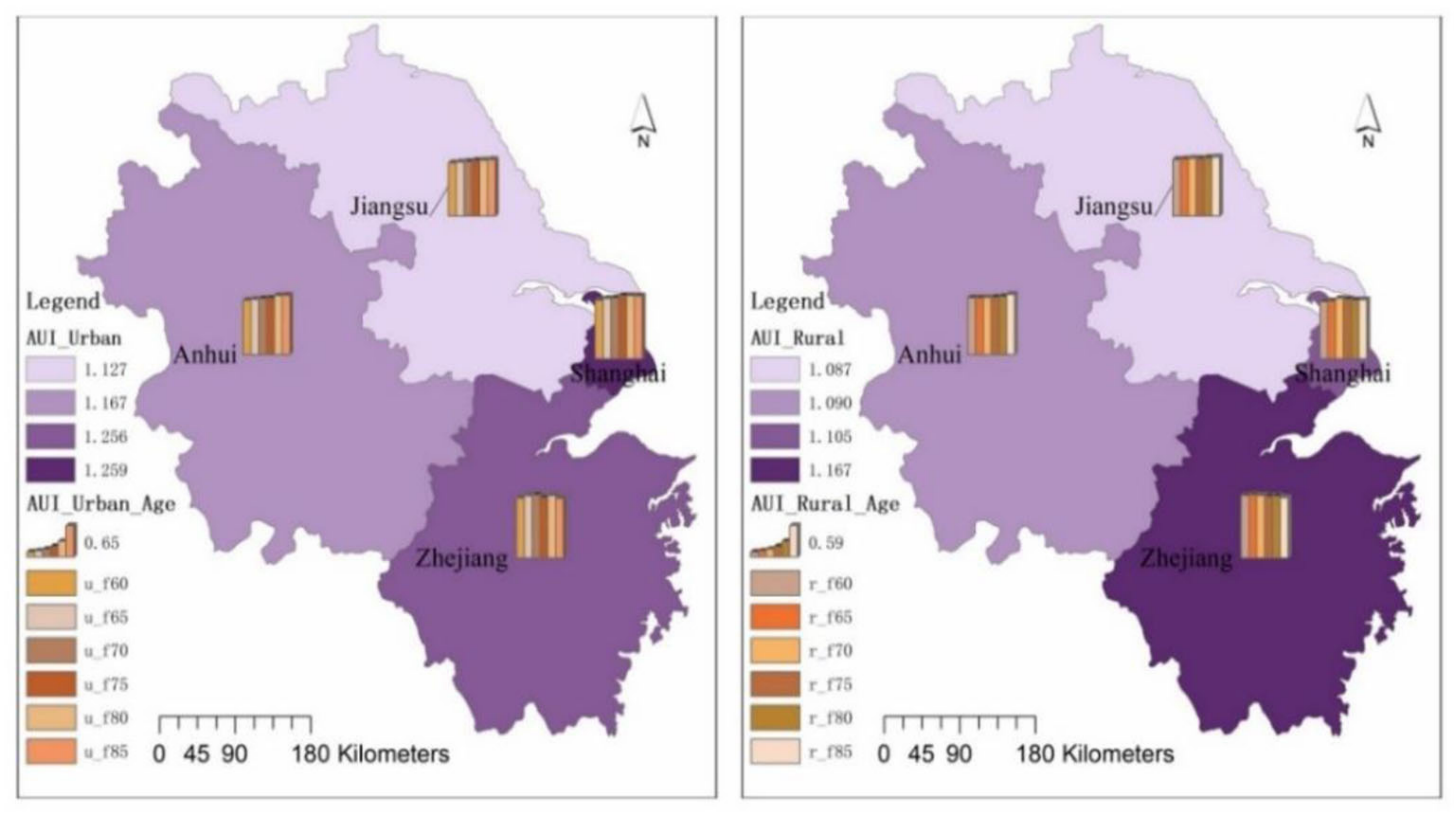
Aids use index for the elderly in urban and rural areas in Jiangsu, Shanghai, Zhejiang and Anhui.

Looking at both urban and rural areas, the frequency of using assistive devices is higher among the urban elderly population in the Yangtze River Delta than among those in rural areas (Figure 7). The urban elderly population in the 60–64 age group, 65–69 age group, 70–74 age group, 75–79 age group and 80–84 age group show an increasing trend in the probability of using assistive devices, but in the 85+ age group, the probability of using assistive devices among the urban elderly population falls back again. Compared to the urban elderly, the frequency of using assistive devices among the elderly population in rural areas shows a fluctuating upward trend, with the probability of using assistive devices among the elderly population in rural areas in the 60–64, 65–69, and 70–74 age groups gradually increasing, then falling back in the 75–79 age group, and the probability of using assistive devices among the elderly population in rural areas in the 80–84 and 85+ age groups rebounded.

In terms of the index of assistive devices used by urban and rural age groups, the index for urban elderly people aged 60–64 was 1.157, with a maximum value of 1.227 (Zhejiang) and a minimum value of 1.091 (Jiangsu); the index for rural elderly people was 1.096, with a maximum value of 1.175 (Zhejiang) and a minimum value of 1.065 (Jiangsu). The Aids Index for the urban elderly was 1.222 at 85+ age group, with a maximum value of 1.288 (Shanghai) and a minimum value of 1.222 (Anhui); the Aids Index for the rural elderly was 1.124, with a maximum value of 1.138 (Anhui) and a minimum value of 1.109 (Shanghai).

In terms of the scores for each provincial administrative unit, relatively speaking, the frequency of use of aids for the urban and rural elderly is highest in Zhejiang in the 60–74 age group, highest in Shanghai in the 75–84 age group and in the 85+ age group, and relatively low in Anhui in the urban and rural elderly.

Comparing the coefficients of variation of each sub-indicator for the use of assistive devices for the elderly in Jiangsu, Shanghai, Zhejiang and Anhui (Table 6).

**Table 6:** Coefficient of variation for indicators of aids use.

**Region**	**Type**	**PG**	**HA**	**UoD**	**UoC**	**UoW**	**BPM**	**BGM**	**CP**	**MI**	**SW**	**CB**	**OA**
JS	U & R	0.064	0.013	0.085	0.159	0.033	0.011	0.004	0.016	0.003	0.000	0.005	0.001
SH	U & R	0.097	0.025	0.160	0.133	0.060	0.061	0.018	0.028	0.009	0.003	0.008	0.001
ZJ	U & R	0.184	0.017	0.100	0.172	0.026	0.040	0.006	0.008	0.018	0.003	0.001	0.008
AH	U & R	0.062	0.014	0.057	0.270	0.077	0.013	0.001	0.017	0.003	0.000	0.005	0.002
YRD	U & R	0.082	0.015	0.080	0.182	0.042	0.013	0.003	0.016	0.005	0.001	0.004	0.002
JS	Urban	0.059	0.016	0.099	0.153	0.037	0.019	0.005	0.020	0.004	0.001	0.008	0.001
SH	Urban	0.086	0.027	0.178	0.135	0.064	0.067	0.017	0.030	0.011	0.004	0.009	0.001
ZJ	Urban	0.187	0.028	0.119	0.178	0.033	0.066	0.009	0.013	0.022	0.004	0.003	0.011
AH	Urban	0.064	0.018	0.106	0.239	0.157	0.023	0.006	0.019	0.006	0.001	0.006	0.004
YRD	Urban	0.076	0.021	0.109	0.170	0.059	0.022	0.004	0.020	0.006	0.001	0.006	0.003
JS	Rural	0.074	0.009	0.065	0.167	0.029	0.009	0.005	0.010	0.002	0.001	0.002	0.003
SH	Rural	0.157	0.015	0.075	0.120	0.040	0.054	0.029	0.018	0.005	0.000	0.000	0.000
ZJ	Rural	0.184	0.006	0.086	0.164	0.017	0.025	0.003	0.002	0.014	0.006	0.002	0.006
AH	Rural	0.082	0.010	0.032	0.302	0.021	0.004	0.004	0.015	0.003	0.000	0.004	0.001
YRD	Rural	0.098	0.008	0.044	0.202	0.023	0.010	0.003	0.010	0.004	0.002	0.001	0.002

At an overall level, the coefficients of variation for indicators of assistive devices such as the use of crutches, the use of presbyopia glasses, the use of dentures and the use of wheelchairs are relatively large for elderly people in Jiangsu, Shanghai, Zhejiang and Anhui. Indicators where the degree of variation in the use of assistive devices among elderly people is in the middle include the use of hearing aids, the use of blood pressure monitors and the use of nursing pads, while indicators where the coefficients of variation are relatively small include the use of blood glucose meters, the use of massage devices, the use of smart wear, the use of nursing beds and the use of other assistive devices.

At the city level, urban elderly people in Jiangsu, Shanghai, Zhejiang and Anhui have relatively large coefficients of variation in indicators of assistive devices such as the use of crutches, the use of dentures, the use of presbyopia glasses and the use of wheelchairs. Indicators in the middle of the range of variation in the use of assistive devices include hearing aids, blood pressure monitors and the use of nursing pads, while other indicators such as smart wear have smaller coefficients of variation.

At the rural level, rural elderly people in Jiangsu, Shanghai, Zhejiang and Anhui have relatively large coefficients of variation in indicators such as use of presbyopia glasses, use of dentures, use of crutches, and use of wheelchairs; the degree of variation in indicators such as use of hearing aids, use of blood pressure monitors, and use of nursing pads is in the middle of the range, while the coefficients of variation in indicators such as use of blood glucose meters, use of massage devices, smart wear, nursing beds, and other assistive devices are smaller.

Looking at the use of assistive devices from the perspective of urban-rural differences, the difference in the use of assistive devices among the urban elderly in the Yangtze River Delta region is higher than that of the rural elderly, as shown by the higher coefficient of variation of indicators such as the use of presbyopia glasses, the use of crutches and smart wear in the rural areas than in the cities, while the coefficient of variation of most assistive devices such as the use of hearing aids, dentures, wheelchairs, blood pressure monitors, blood glucose meters, nursing pads, massage devices, nursing beds and other assistive devices is higher in the urban elderly than in the rural elderly.

## Analysis of factors influencing healthcare needs among elderly population

The ability to eat on their own, bathe on their own, get in and out of bed and other activities of daily living, incontinence and the use of assistive devices are all physical indicators (or objective indicators) of healthcare for elderly people, while the need for care for elderly people is a subjective description of their own care needs. This section will analyze the relationship between the elderly healthcare needs and the physical indicators, not only by correlating the subjective and objective indicators of elderly people's health needs, but also by analyzing the relationship between economic and social factors and elderly people's healthcare needs (Table 7).

**Table 7:** Analysis related to the health care needs of the elderly.

**Units**	**R-value**	**Significance**	**Units**	**R-value**	**Significance**
Eating	0.933	0.01	Dentures	0.183	Not sig
Dressing	0.982	0.01	Crutches	0.927	0.01
Go to the toilet	0.951	0.01	Wheelchair	0.819	0.01
Get in and out of bed	0.958	0.01	Sphygmomanometer	−0.12	Not sig
Walking around indoors	0.882	0.01	Blood Glucose Meter	−0.118	Not sig
Bathing	0.987	0.01	Care pads	0.935	0.01
Fecal incontinence	0.903	0.01	Massage equipment	−0.348	Not sig
Urinary incontinence	0.961	0.01	Smart Wear	−0.227	Not sig
No incontinence	0.954	0.01	Nursing beds	0.906	0.01
Presbyopic glasses	−0.467	0.05	Other Aids	0.064	Not sig
Hearing aids	0.894	0.01			

### Analysis of physical factors in the demand for healthcare among elderly population

#### Analysis of the correlation between healthcare needs of elderly and daily activities

**Figure 8:**
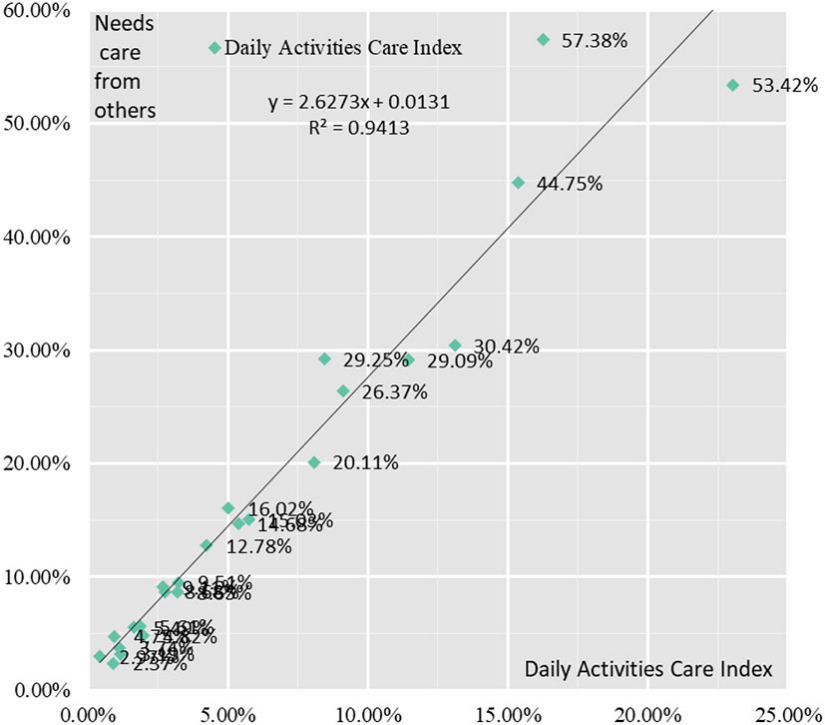
Correlation analysis between healthcare needs of the elderly and the need for daily care activities.

The proportion of elderly people in need of care in the YRD increases gradually with age. The proportion of care needs for the 60–64, 65–69, 70–74, 75–79, 80–84, and 85+ age groups are 3.76, 5.75, 9.49, 14.91, 24.84, and 45.52% for each age groups, respectively. The average of the proportion of each indicator of care for daily activities for the 60–64, 65–69, 70–74, 75–79, 80–84, and 85+ age groups was 1.13, 1.83, 3.25, 5.70, 8.82, and 16.78%, respectively. The proportion of elderly people in need of care and the proportion of each indicator of daily activities care in the Yangtze River Delta region show a development in the same direction, that's, the relationship between the two becomes closer as the elderly grow elderly. Similarly, when the proportion of elderly people in need of healthcare and the proportion of each indicator of daily activities care in urban and rural areas of Jiangsu, Shanghai, Zhejiang and Anhui in the Yangtze River Delta region were correlated, they had a strong positive correlation with a correlation coefficient of 0.97 (Figure 8).

Correlation analysis of the indicators of daily activities care with the proportion of elderly people requiring care showed that there was still a strong positive correlation between each indicator and the proportion of elderly people requiring care. The correlation coefficients between the proportion of older people with difficulties eating, dressing, toileting, getting in and out of bed, walking indoors, bathing and the proportion of older people in need of care were 0.933, 0.98, 0.95, 0.96, 0.88, and 0.99, respectively.

#### Analysis of the correlation between healthcare needs of elderly and incontinence

**Figure 9:**
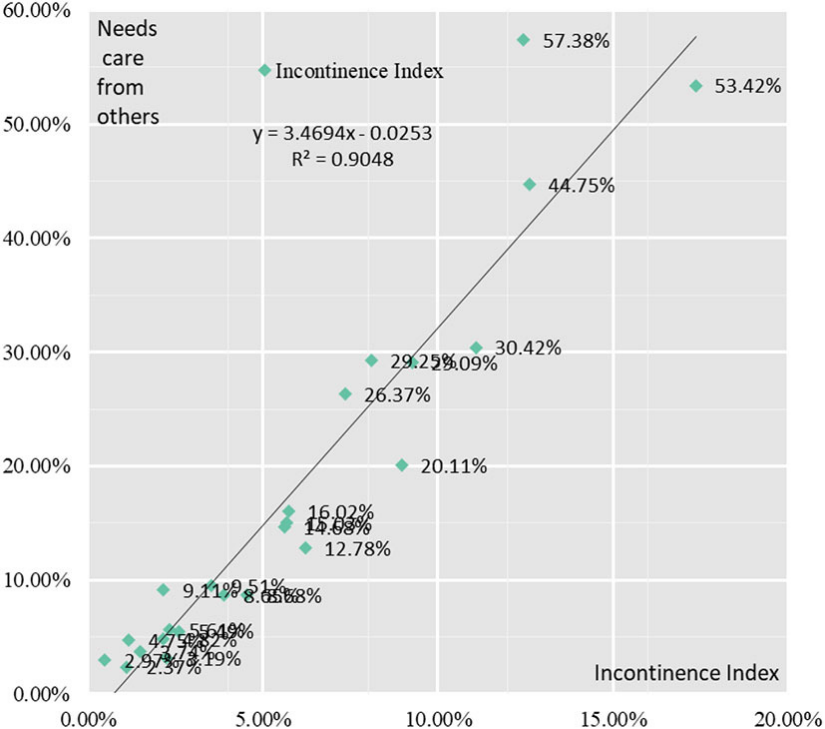
Correlation analysis between healthcare needs of elderly people and incontinence.

The probability of incontinence among elderly people in the YRD increases gradually with age. The proportion of elderly people with incontinence in the 60–64, 65–69, 70–74, 75–79, 80–84, and 85+ age groups were 1.58, 2.79, 3.95, 6.14, 7.58, and 13.48%. The proportion of elderly people in need of care and the proportion of incontinence in the Yangtze River Delta show the same direction, that's the relationship between the two becomes closer as elderly people age. Similarly, when the proportion of elderly people in need of care and the proportion of incontinence in urban and rural areas of Jiangsu, Shanghai, Zhejiang and Anhui in the Yangtze River Delta region were correlated, they had a strong positive correlation with a correlation coefficient of 0.95 (Figure 9).

Correlation analysis of the three indicators in the Incontinence Index with the proportion of elderly people in need of care showed that there was still a strong positive correlation between each indicator and the proportion of elderly people in need of care. The correlation coefficients between the proportion of elderly people with fecal incontinence, urinary incontinence and incontinence and the proportion of elderly people in need of care were 0.90, 0.96, and 0.95, respectively.

#### Analysis of the correlation between healthcare needs of elderly and aids use

**Figure 10:**
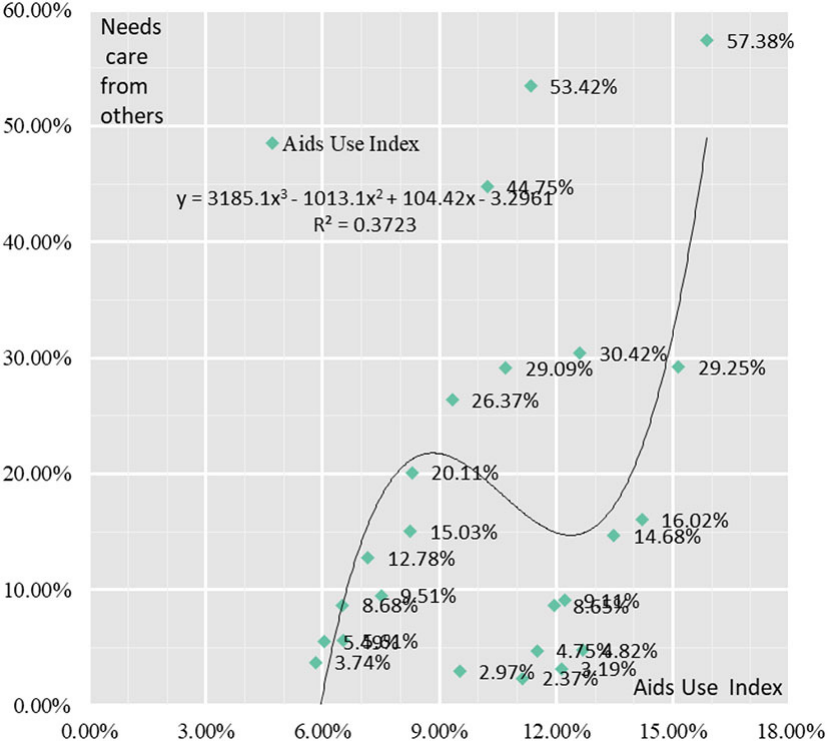
Correlation analysis between healthcare needs of elderly people and Aids Use.

The use of Aids devices for the elderly in the Yangtze River Delta region as a whole shows a gradual increase with age. The proportion of the 60–64 age group, 65–69 age group, 70–74 age group, 75–79 age group, 80–84 age group and 85+ age group using assistive devices are 7.58, 8.42, 8.87, 9.68, 11.44, and 11.85%, respectively. The correlation between the proportion of elderly people in need of care and the proportion of aids used in urban and rural areas of Jiangsu, Shanghai, Zhejiang and Anhui in the Yangtze River Delta region was found to be not a linear correlation but a cubic function curve correlation, with a strong correlation coefficient of 0.61 (Figure 10).

A correlation analysis of the indicators in the Assistive Devices Use Index with the proportion of elderly people in need of care showed that there were significant differences in the correlation between the indicators and the proportion of elderly people in need of care. There were significant positive correlations between the proportion of elderly people using hearing aids, the proportion of crutches, the proportion of wheelchairs, the proportion of nursing mats and the proportion of nursing beds and the proportion in need of care, with correlation coefficients of 0.89, 0.93, 0.82, 0.94, and 0.91, respectively. There was negative correlation between the proportion of elderly people using presbyopia glasses, massage devices, blood pressure monitors, blood glucose meters and smart wear and the proportion requiring care, with their correlation coefficients being – 0.47, – 0.35, – 0.12, – 0.12, and – 0.23, respectively. The correlation coefficient between the proportion of elderly people using and the proportion requiring care was 0.06, with a weak correlation strength.

### Analysis of economic and social factors in the demand for healthcare among elderly population

#### Medical conditions

Medical conditions are fundamental to the healthcare of elderly people. The quality of healthcare conditions is directly related to the quality of healthcare for the elderly. The Yangtze River Delta region has relatively excellent medical and healthcare resources and is able to provide high quality medical services for elderly healthcare services, providing a guarantee for the treatment and diagnosis of elderly diseases and health check-ups. Compared to the basic medical conditions in other provinces and cities across China, the total number of hospitals in the Yangtze River Delta region is 5,214, the total number of hospital beds is 120,2791 and the number of practicing doctors is 728,189, accounting for 15.24, 17.59, and 18.69% of the national total, respectively. in 2020, the number of practicing doctors in the Yangtze River Delta cities of Shanghai, Hangzhou, Nanjing, Suzhou, Ningbo and Wenzhou 78,364, 51,135, 37,823, 37,185, 31,891, and 31,472, respectively, accounting for six of the 25 cities with the highest number of occupational physicians nationwide. Among the 25 cities with the highest number of hospital beds nationwide, cities in the Yangtze River Delta region accounted for five of them, namely Shanghai, Hangzhou, Suzhou, Hefei and Nanjing, corresponding to 143,638, 84,251, 63,611, 59,271, and 57,455 hospital beds, respectively, with the number of hospital beds in Shanghai, Hangzhou, Suzhou, Hefei and Nanjing accounting for 3.69, 2.16, and 1.63% of the national proportion, respectively, 2.16, 1.63, 1.52, and 1.47%, respectively, for a total of 10.48%.

#### Community services for elderly people

Aging in place and community-based care together form an integral part of China's retirement protection. While aging at home is currently the preferred option for the majority of China's elderly, the importance of aging in the community is becoming increasingly prominent in the long term. The survey shows that in the Yangtze River Delta region, elderly people are more “home-loving” as their age increases, with 75.16% of the 60–64 age group choosing to age at home and 81.58% of the 85+ age group choosing to age at home. The proportion of those who chose to age in an institution fell from 6.34 to 3.87% (Table 8). The proportion of elderly people in the Yangtze River Delta choosing to receive care and attention from friends or neighbors, volunteers, home helpers, people from medical care institutions and people from aged-care institutions is low, with only 1.45% of the 60–64 age group being cared for by aged-care institutions and home helpers, etc., and the proportion of elderly people in the 85+ age group receiving care and attention in the community gradually increasing to 12.71% (Table 9).

**Table 8:** Willingness of elderly people in the Yangtze River Delta region to age in a residential care facility.

**Age group**	**At Home (%)**	**In the community during the**	**In a nursing**	**Depending on**
		**day and home at night (%)**	**home (%)**	**the situation (%)**
60–64	75.16	2.63	6.34	15.87
65–69	76.25	2.15	5.98	15.62
70–74	78.76	2.26	5.04	13.94
75–79	80.56	2.00	5.08	12.36
80–84	78.23	8.43	4.26	9.08
85+	81.58	8.36	3.87	6.20

**Table 9:** Proportion of elderly groups in the Yangtze River Delta with community-based care and attention.

**Age group**	**Neighbors (%)**	**Volunteers (%)**	**Housekeeping staff [nannies**,	**Healthcare facility**	**Staff in elderly**	**Total (%)**
			**hourly workers] (%)**	**staff (%)**	**institutions (%)**	
60–64	0.24	0.00	0.97	0.24	0.00	1.45
65–69	0.80	0.00	0.80	0.00	0.60	2.20
70–74	0.00	0.00	1.67	0.17	1.00	2.84
75–79	0.28	0.00	3.20	0.00	2.09	5.56
80–84	0.23	0.11	6.50	0.23	2.05	9.12
85+	0.28	0.37	9.76	0.55	1.75	12.71

**Figure 11:**
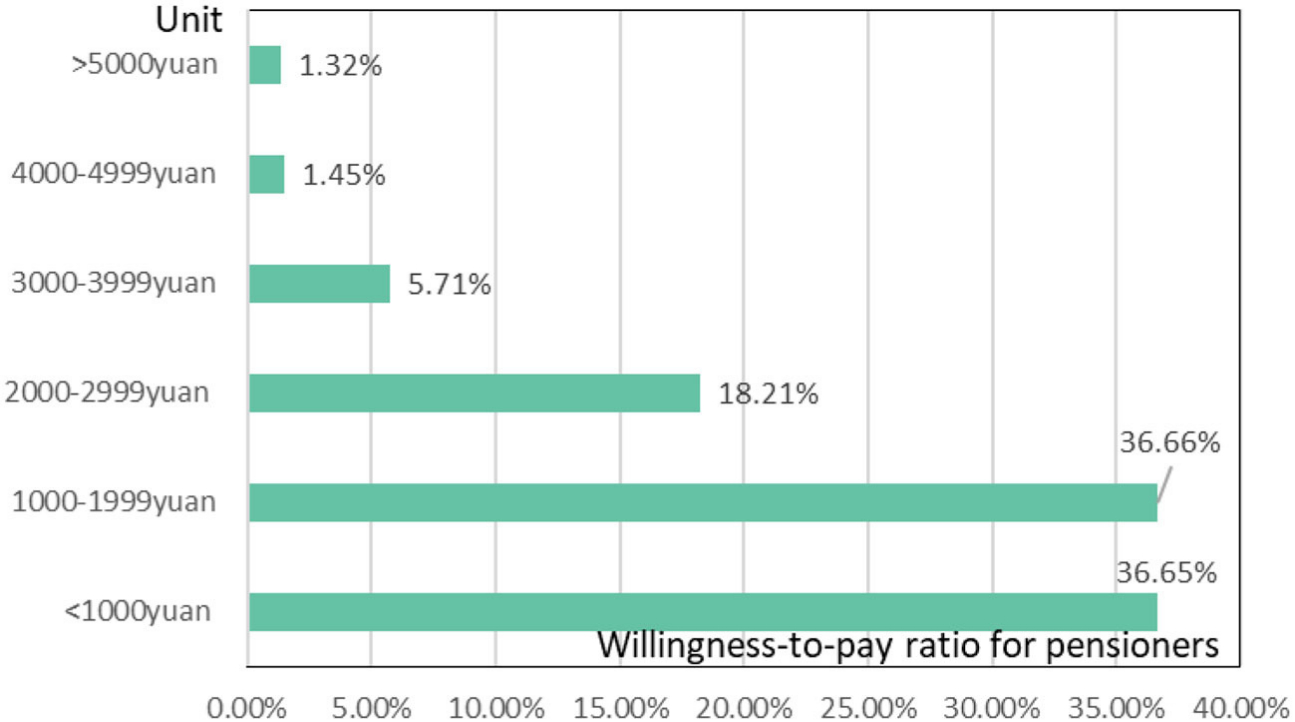
Elderly people's willingness to pay for a place in a residential care facility.

In the light of the growing trend of aging and childlessness in China, community-based care will form an important vehicle for the healthcare of China's elderly population for a long time to come. At this stage, the low rate of institutionalization of elderly people in China is due to a combination of factors. Firstly, there are economic factors. Most elderly people pay less than RMB 2,000/month for a nursing home. 36.65% of elderly people in the Yangtze River Delta are willing to pay less than RMB 1,000/month for a nursing home, 36.66% are willing to pay RMB 1,000–1999/month and 18.21% are willing to pay RMB 2,000–2,999/month (Figure 11). Relative to the willingness of the elderly to pay for admission to a retirement institution, the cost of admission to both public and private retirement institutions is well over RMB2,000/month, and the lack of payment by the elderly in retirement is an economic factor that leads to the low proportion of admission to a retirement institution. The second is the quality of elderly care services. Insufficient supply of elderly service personnel and an elderly age structure directly affect the quality of elderly care services, and to a certain extent, the choice of elderly institutions for healthcare. Take Shanghai as an example, Shanghai's elderly care workers are mainly middle-aged people aged 50–59, accounting for 62.24%. The proportion of those with primary certificates (including medical care and healthcare) for elderly care workers is the highest, accounting for 69.09%, while the proportion of those with intermediate certificates for elderly care workers is 7.24%, while < 1% have senior, technician and senior technician certificates for elderly care workers, and 22.36% of elderly care workers are uncertified, indicating that vocational training for elderly care needs to be further strengthened. Once again, there is the cultural factor. For a long time, China has had a cultural tradition of “bring up children for the purpose of being looked after in old age”, meaning that when one reaches old age, one relies mainly on one's children for old age and is blessed with many children. However, the reality is that as the problem of childlessness becomes more and more prominent, the current Chinese elderly are mostly assisted by their children in healthcare and care, and it is becoming less and less likely that the middle-aged, that's, the future Chinese elderly, will rely on their children to assist them in their old age, meaning that the traditional cultural factor of “bring up children for the purpose of being looked after in old age” will eventually be broken, and new community-based, home-based and institutional care will be introduced.

#### Level of education

The digital age has seen the development of intelligent use of healthcare for the elderly. At the same time, the digital divide in terms of access to transport, medical care and shopping is still prominent for many elderly groups due to their low level of education. The proportion of elderly people in the Yangtze River Delta region who have not attended school (including literacy classes), primary school (including private schools), junior high school, senior high school, university specialist and bachelor's degree or higher education are 39.78, 33.44, 16.23, 6.96, 2.23, and 1.36%, respectively, meaning that nearly seven layers of elderly people in the Yangtze River Delta region have < 6 years of education. The correlation analysis between the proportion of elderly people in need of care in urban and rural areas of Jiangsu, Shanghai, Zhejiang and Anhui in the Yangtze River Delta and the proportion of elderly people who had not attended school, primary school (including private school), junior high school, high school/junior college/vocational high school, undergraduate level, and bachelor's degree and above, respectively, showed correlation coefficients of 0.67, – 0.43, – 0.54, – 0.30, – 0.18, and 0.02, where the elderly age group who had not attended school increased in need of care as they got older.

#### Marital status

A good marital situation is one of the necessary conditions for a high quality of life in old age. Good marital status includes at least two aspects: harmony in the family and good health for both spouses. Statistics on the marital structure of elderly people in the Yangtze River Delta region show that 63.37, 32.46, 2.84, and 1.33% are spouses, widowed, divorced and never married, respectively. The correlation coefficients between the proportion of elderly people in need of care and the proportion of spouses, widowed, divorced and never married were – 0.89, 0.92, – 0.11, and – 0.39 for the urban and rural areas of Jiangsu, Shanghai, Zhejiang and Anhui, respectively, and the correlation coefficients reflected a strong negative correlation between the willingness of elderly people with spouses to need care (from others) and the willingness of elderly people who were widowed to need care (from others). This indicates that marital status has an impact on the need for healthcare in old age. According to our statistics, the proportion of elderly people in the Yangtze River Delta who were cared for by their spouses was 79.42, 75.00, 69.06, 52.99, 31.58, and 11.23% in the 60–64, 65–69, 70–74, 75–79, 80–84, and 85+ age groups, respectively. The proportion of the above different age groups who were cared for by their children (including sons, daughters-in-law, daughters, sons-in-law and grandchildren) were 15.50, 19.00, 25.75, 40.19, 58.72, and 74.95%, respectively. In other words, healthcare for the young and old group (60–75 years old) is mainly provided by spouses, but for the middle aged (75–84 years old) and the old (85 years old and above) it is mainly provided by children. This means that a good marital status is crucial for the elderly Chinese group up to the age of 75 in their later years.

## Discussion and conclusions

### Discussion

This article presents a comprehensive evaluation of the differences in the healthcare needs of the elderly population in the deeply aging and economically developed Yangtze River Delta region of China. An attempt was made to construct a comprehensive index of healthcare in three dimensions: daily activities care, incontinence care and use of assistive devices, which will be useful for the study of healthcare for the elderly.

Where our study differs from the findings of Wang's study is (42): first, where there is common ground. Both studies agree that China's population is aging and that there is a lack of healthcare for the elderly. The second area of difference is that Wang's study points to the difficulty of meeting the diverse needs of elderly people for home healthcare services due to the low utilization of resources and uneven development of healthcare services. We explain the impact on healthcare services for the elderly from the perspective of community aged care services and point out the reasons for the current low utilization of community aged care services.

In addition, our study complements Wang B.O. (43) in the empirical analysis section, we point out that there are significant differences in the use of assistive devices among the elderly population of different ages and regions, especially in the use of assistive devices such as smart wear, which are hardly used by the elderly population in rural areas, but we have yet to explain the reasons for the low use of assistive devices such as smart wear among the elderly in the higher age groups. In contrast, Wang B. explained the willingness of elderly people to use smart aging products from the perspective of emotional attachment, which corresponds to our study.

### Conclusions

This article Taking the Yangtze River Delta region of China as an example, constructs a comprehensive healthcare index to explore the healthcare needs of the elderly and the factors influencing them, and draws the following key conclusions.

(1) The overall picture shows a gradual increase in the level of healthcare needs of the elderly population. The results of the empirical analysis show that 75 years of age is the cut-off point for the health care needs of the elderly. The three age groups of 60–64, 65–69, and 70–74 years of age have a relatively moderate increase in health care needs, with the Healthcare Composite Index increasing from 3.162 to 3.264, a growth rate of 3.25%. However, the health care needs of older people in the 75–79, 80–84, and 85+ age groups grew relatively quickly, with the Healthcare Composite Index increasing from 3.370 to 3.797, a growth rate of 12.67%.
(2) The difference in scores for the secondary indicators shows that Anhui has the largest Daily Activities Care Index and Incontinence Index, while Zhejiang has the smallest Daily Activities Care Index and Incontinence Index. The use of assistive devices was highest among elderly people in Shanghai and lowest among elderly people in Jiangsu. In terms of overall differences in secondary indicator scores, the greatest differences were found in the Daily Activities Index and Incontinence Index for elderly people in Anhui, and the smallest differences were found in the daily activities index and incontinence index for elderly people in Zhejiang. The coefficients of variation for indicators of assistive devices such as the use of crutches, the use of presbyopia glasses, the use of dentures and the use of wheelchairs were relatively large for elderly people in the Yangtze River Delta, while the coefficients of variation for indicators such as the use of blood glucose meters, the use of massage devices, the use of smart wear, the use of nursing beds and the use of other assistive devices were relatively small.
(3) In terms of differences in the scores of the three-level indicators, the differences in the three-level indicators of healthcare needs are greater between urban and rural areas. In terms of daily activities care and incontinence index, the difference in scores for the three-level indicators is higher in urban than in rural areas in Jiangsu, Zhejiang, Shanghai and Anhui; in terms of the use of assistive devices, the coefficient of variation for most assistive devices, such as hearing aid use, denture use, wheelchair use, blood pressure monitor use, blood glucose meter use, nursing pad use, massage appliance use, nursing beds and other assistive tools, is higher in urban than in rural areas, and in rural areas The coefficients of variation for the indicators of presbyopia use, crutches use and smart wear were higher for elderly people in rural areas than in urban areas.
(4) The demand for elderly healthcare is highly positively correlated with the daily activities care index and incontinence index, with Pearson correlation coefficients of 0.97 and 0.95, respectively; the demand for elderly healthcare and the use of assistive devices index show a cubic curve correlation, with a correlation coefficient of 0.61. The strength of the correlation between the demand for elderly healthcare and some sub-indicators of the use of assistive devices is weak. The correlation coefficient is 0.61.
(5) Medical conditions are macro conditions that affect the demand for healthcare for the elderly. Excellent medical and health conditions provide a guarantee for healthcare for the elderly in the Yangtze River Delta. Community aged care services are a meso condition affecting the healthcare needs of the elderly, but the low proportion of elderly people staying in aged care institutions is due to a combination of low elderly payment ability, general quality of aged care services and cultural factors. Educational attainment and marital status are micro conditions that influence the demand for healthcare in old age. In terms of educational attainment, elderly people who have not attended school have an increasing need for care as they get older. In terms of marriage, there is a strong negative correlation between the willingness to need healthcare among older people with a spouse and a strong willingness to need care among older people who are widowed.

## Data availability statement

The datasets are publicly available via China National Committee on Ageing. Dataset of the Fourth Sample Survey on the Living Conditions of China's Urban and Rural Older Persons. Hualing Press: Beijing, China (2020).

## Author contributions

Conceptualization and supervision: JW. Methodology and writing—original draft preparation: CL. Data collection: YL. Writing—review and editing: YH. All authors contributed to the article and approved the submitted version.

## Funding

National Social Science Foundation of China Research on the Policy Implementation Ability of County-level Government (Grant Number 19CZZ039); Shanghai Municipal Student Innovation Training Program Research on the evaluation and optimization of urban community elderly service quality based on UPE methods (Grant Number cs2203017); The Fundamental Research Funds for the Central Universities Study on multi-objective coordination of rural green development in the perspective of Rural Revitalization (Grant Number 2232022E-07).

## Conflict of interest

The authors declare that the research was conducted in the absence of any commercial or financial relationships that could be construed as a potential conflict of interest.

## Publisher's note

All claims expressed in this article are solely those of the authors and do not necessarily represent those of their affiliated organizations, or those of the publisher, the editors and the reviewers. Any product that may be evaluated in this article, or claim that may be made by its manufacturer, is not guaranteed or endorsed by the publisher.

## Abbreviations

DCI: Daily Activities Care Index
ICI: Incontinence Index
AUI: Aids Use Index
HCCI: Healthcare Composite Index
U&R: Urban and Rural
JS: Jiangsu
SH: Shanghai
ZJ: Zhejiang
AH: Anhui
YRD: Yangtze River Delta Region
PG: Presbyopia glasses
HA: Hearing aids
UoD: Use of Dentures
UoC: Use of Crutches
UoW: Use of Wheelchairs
BPM: Blood Pressure Monitors
BGM: Blood Glucose Meters
CP: Care Pads
MI: Massage Instruments
SW: Smart Wear
CB: Care Beds
OA: Other Aids.
